# Long-Term Trends in Laryngeal Cancer Incidence and Mortality in Central Serbia (1999–2023): A Joinpoint Regression Analysis

**DOI:** 10.3390/healthcare13131633

**Published:** 2025-07-07

**Authors:** Vladimir Nešić, Dragana Krstić Nešić, Sandra Šipetić Grujičić, Bojana Bukurov, Dragan Miljuš, Snežana Živković Perišić, Aleksandra Nikolić

**Affiliations:** 1 Faculty of Medicine, University of Belgrade, 11000 Belgrade, Serbia; dkrstic75@gmail.com (D.K.N.); sandra.grujicic@med.bg.ac.rs (S.Š.G.); bojana.bukurov@med.bg.ac.rs (B.B.); aleksandra.nikolic@med.bg.ac.rs (A.N.); 2 Clinic of Otorhinolaryngology and Maxillofacial Surgery, University Clinical Centre of Serbia, 11000 Belgrade, Serbia; 3 Institute of Epidemiology, Faculty of Medicine, University of Belgrade, 11000 Belgrade, Serbia; 4 Institute of Public Health of Serbia “Dr Milan Jovanović Batut”, 11000 Belgrade, Serbia; dragan_miljus@batut.org.rs (D.M.); snezana_zivkovic@batut.org.rs (S.Ž.P.)

**Keywords:** laryngeal cancer, incidence, mortality, epidemiology, trends, Central Serbia

## Abstract

**Background/Objectives:** Laryngeal cancer (LC) accounts for 1–3% of all malignant neoplasms. The aim of this study was to analyze temporal trends in the incidence and mortality of LC in Central Serbia over a 25-year period (1999–2023). **Methods:** Data on newly diagnosed cases and deaths, stratified by sex and age group, were obtained from the Serbian Cancer Registry. Crude, age-specific, and age-standardized incidence and mortality rates were calculated. Joinpoint regression analysis was used to estimate average annual percent changes (AAPCs) and assess their statistical significance. **Results:** The average annual age-standardized incidence rate (ASR-W) was 11.1 per 100,000 in men and 1.4 in women, with corresponding mortality rates of 5.4 and 0.5, respectively. The highest incidence was observed in the 60–69 age group for both sexes (61.1/100,000 in men; 7.4/100,000 in women), while the highest mortality was recorded in individuals aged ≥70 (35.7/100,000 in men; 3.8/100,000 in women). A statistically significant annual decline among men was observed in both incidence (ASR-W: −0.7%) and mortality (ASR-W: −2.0%). In contrast, trends among women were not statistically significant, indicating overall stability. **Conclusions:** Although the Cancer Registry in Serbia faces limitations primarily due to data quality issues, it is a key tool for understanding LC trends, guiding health policies, and effectively allocating resources. Given the substantially higher burden among men, it is essential to strengthen tobacco and alcohol control, improve occupational safety, and promote early detection and timely treatment to reduce the disease burden.

## 1. Introduction

Laryngeal cancer (LC) accounts for approximately 1–3% of all malignant tumors and represents the second most frequently diagnosed malignancy within the head and neck region [1]. Squamous cell carcinoma (SCC) accounts for over 90% of all malignant tumors of the larynx, others include verrucous carcinoma, carcinosarcoma, basaloid squamous cell carcinoma, lymphoepithelial carcinoma and connective tissue tumors (e.g., chondrosarcoma, fibrosarcoma, leiomyosarcoma, rhabdomyosarcoma, and angiosarcoma), as well as secretory tumors (neuroendocrine and minor salivary gland tumors), lymphoproliferative tumors, melanomas, and metastatic tumors [2,3,4].

LC remains a significant global health concern. According to GLOBOCAN estimates, 189,191 new cases were reported worldwide in 2022, corresponding to an age-standardized incidence rate to the world standard population (ASR-W) of 1.9 per 100,000 [5]. LC ranks as the 20th most common malignancy globally, accounting for approximately 1% of all cancer cases, with ASR-Ws of 3.5 per 100,000 in men and 0.4 in women [6]. In terms of mortality, LC was responsible for an estimated 103,359 deaths worldwide in 2022, with a global ASR-W of 1.0 per 100,000. It ranked 18th among all malignancies and accounted for approximately 1% of cancer-related deaths. The corresponding ASR-Ws were 1.9 per 100,000 in men and 0.2 in women [6].

The incidence and mortality rates of LC vary significantly by region, influenced by factors such as tobacco and alcohol use, occupational exposures, and socioeconomic status. Although LC incidence and mortality generally declined in most countries between 1980 and 2017, likely due to a decline in smoking and alcohol consumption, these downward trends were less evident among females, younger populations, and countries within the European region [7,8,9].

Despite advancements in medical technology and early detection, the burden of LC remains high, particularly in low- and middle-income countries, where access to healthcare is limited [10]. Disparities in healthcare infrastructure and preventive measures lead to delayed diagnoses and suboptimal treatment outcomes in these regions. Limited access to treatment modalities in some regions may contribute to higher mortality rates [7].

In Central Serbia, comprehensive population-based research on long-term trends in LC incidence and mortality is still lacking, so this study aimed to analyze LC incidence and mortality in Central Serbia between 1999 and 2023 and to give comprehensive analyses of long-term trends.

## 2. Materials and Methods

### 2.1. Study Setting and Population

**Figure 1:**
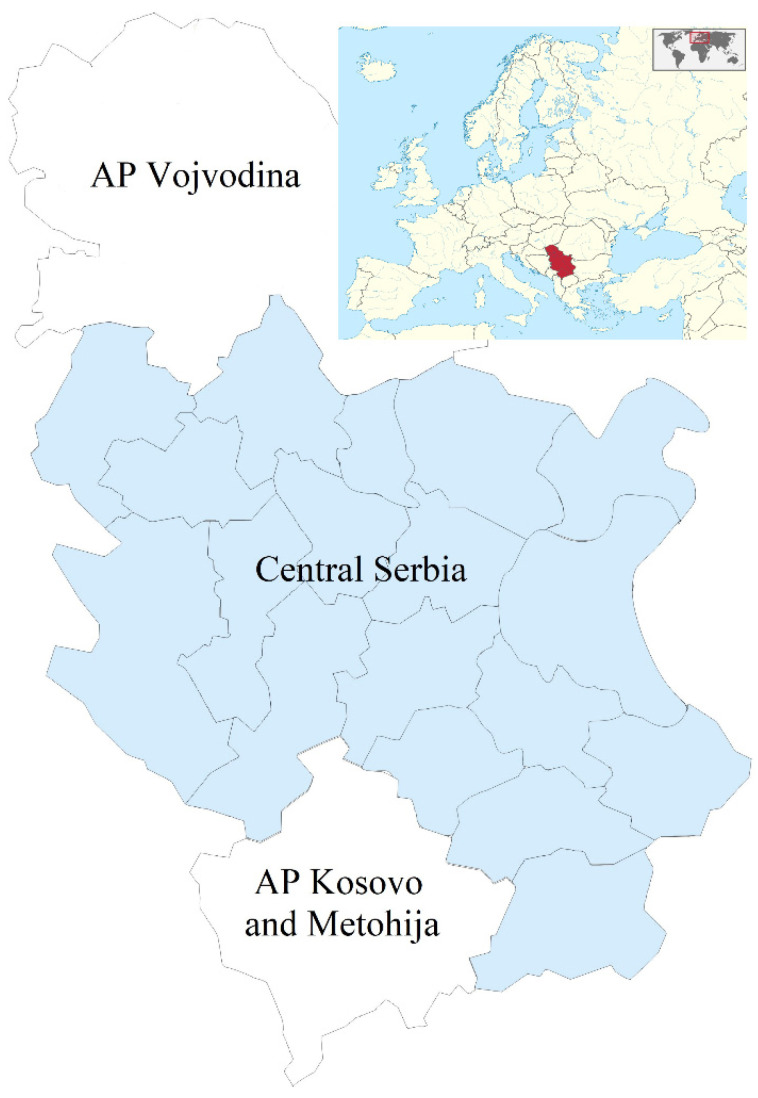
Map of the Republic of Serbia with Autonomous Provinces (AP). Central Serbia within the Republic of Serbia.

Central Serbia refers to the part of the Republic of Serbia that excludes the autonomous provinces of Vojvodina and Kosovo and Metohija [Figure 1]. According to the 2022 census, Central Serbia had 4,906,773 inhabitants (2,386,239 men and 2,520,534 women) [11].

### 2.2. Data Sources

Data on newly diagnosed LC cases and related deaths between 1999 and 2023 were obtained from the Cancer Registry of Central Serbia, maintained by the Institute of Public Health of Serbia, “Dr Milan Jovanović Batut” [12,13]. The Registry operates under legal mandate [14] and was reorganized in 1996 in accordance with recommendations from the International Agency for Research on Cancer (IARC) and the European Network of Cancer Registries (ENCR). Since 1998, it has been a member of both IACR and ENCR [15,16].

Cancer reporting in Serbia is mandatory, and the Registry systematically collects data from all healthcare levels, including outpatient and hospital services, pathology labs, and private institutions. These data sources are integrated to enhance completeness and accuracy. Quality assurance is based on international standards and includes regular evaluation of coverage and validity using indicators such as the proportion of pathologically confirmed cases, death certificate-only (DCO) cases, and unspecified primary tumor sites. Mortality-to-incidence ratio and external audits are used to assess underregistration.

Annual population estimates and censuses for Central Serbia in the observed period were obtained from the Statistical Office of the Republic of Serbia [11]. Data for 2023 were directly obtained from the Institute due to lack of public availability. Laryngeal cancer was classified under ICD-10 code C32 [17].

### 2.3. Rate Calculation and Standardization

Crude, specific, and age-standardized incidence and mortality rates were calculated. Age standardization was performed using the direct method, with the world standard population by Segi [18].

### 2.4. Trend Analysis

Temporal trends were assessed using Joinpoint regression analysis applied to age-standardized rates. Temporal trends were analyzed using Joinpoint regression applied to age-standardized rates, allowing for the estimation of annual percentage change (APC) and the detection of statistically significant changes in trend over time. Based on the optimal model selected by the permutation test in the Joinpoint software, the average annual percent change (AAPC) was also calculated. Separate AAPCs were computed for men and women. The independent variable was the year, and the dependent variable was the respective rate. The Grid Search method was used [19]. To assess the statistical significance of the AAPC, 95% confidence intervals (CIs) were calculated.

### 2.5. Comparability Test and Statistical Software

A comparability test was used to compare trends between sexes to determine whether the segmented regression functions were parallel (parallelism test) [20]. All analyses were performed using IBM SPSS Statistics version 21.0 and the Joinpoint Regression Program version 5.4.0 [21,22].

## 3. Results

### 3.1. Position of LC Among Leading Cancer Sites

Among men, LC accounted for an average of 3.6% of the total number of new cancer cases (range: 2.6–4.7%) and 3.3% of cancer-related deaths (range: 2.5–4.5%). Over the 25-year period (1999–2023), LC consistently ranked among the ten leading cancer sites in men in Central Serbia, based on its proportional representation. In contrast, it was not among the top ten in women.

### 3.2. Sex-Specific Incidence and Trends in LC

Between 1999 and 2023, a total of 14,429 individuals were diagnosed with LC in Central Serbia, including 12,636 men and 1793 women. On average, 505 new cases were reported annually in men, corresponding to a crude incidence rate of 19.7 per 100,000 and an ASR-W of 11.1 per 100,000. In women, the average annual number of new cases was 72, with a crude rate of 2.7 per 100,000 and an ASR-W of 1.4 per 100,000 (Table 1). A statistically significant annual decrease in ASR-W for incidence was observed in men (AAPC = −0.7%), while a non-significant increase was recorded in women (AAPC = +0.9%) (Table 2).

### 3.3. Age-Specific Incidence and Trends in LC

The highest age-specific incidence rate in men was recorded in the 60–69 age group, reaching 61.1 per 100,000. In women, the peak age-specific incidence was also observed in the 60–69 group, at 7.4 per 100,000 (Table 3). Statistically significant AAPC increases were observed in the 60–69 and 70+ age groups, with the most pronounced rise of 2.8% per year among women aged 70 and older (Table 2).

### 3.4. Sex-Specific Mortality and Trends in LC

Between 1999 and 2023, a total of 7348 individuals died from LC in Central Serbia, including 6592 men and 756 women. In men, the average annual number of LC-related deaths was 264, with a crude mortality rate of 10.3 per 100,000 and an ASR-W of 5.4 per 100,000. In women, the average annual number of deaths was 30, with a crude mortality rate of 1.1 per 100,000 and an ASR-W of 0.5 per 100,000 (Table 4). Overall, ASR-W mortality rates showed a declining trend in both sexes, with an AAPC of −2.0% in men and −0.7% in women; however, statistical significance was confirmed only in men (Table 5).

### 3.5. Age-Specific Mortality and Trends in LC

The highest age-specific mortality rate in men was observed in the 70+ age group, reaching 35.7 per 100,000 (Table 6). A statistically significant decline in mortality rates was recorded across all male age groups, as indicated by the AAPC: −4.9% per year in the 0–49 age group, −2.5% in 50–59, −1.3% in 60–69, and −0.8% in those aged 70 and older. In contrast, the highest female age-specific mortality rate was also observed in the 70+ age group, at 3.8 per 100,000, with a statistically significant AAPC increase of +1.5% (Table 5).

### 3.6. Sex-Based Comparison of Incidence and Mortality Trends

**Figure 2:**
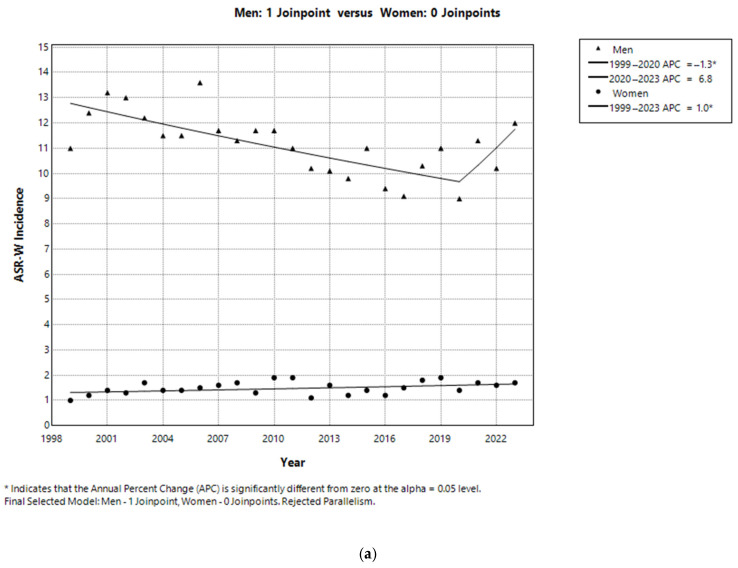

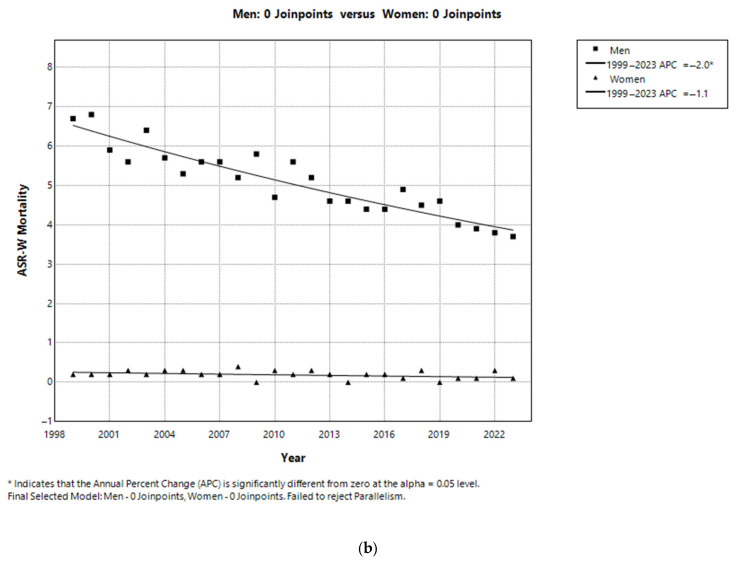
(**a**) Joinpoint regression analysis showing the test of parallelism for ASR-W incidence trends of laryngeal cancer in Central Serbia, 1999–2023. (**b**) Joinpoint regression analysis showing the test of parallelism for ASR-W mortality trends of laryngeal cancer in Central Serbia, 1999–2023.

The comparability test showed that incidence trends were not parallel between men and women (*p* = 0.011; Figure 2a), whereas mortality trends were parallel (*p* = 0.171; Figure 2b).

## 4. Discussion

### 4.1. Incidence and Mortality Rates

According to our findings, the average ASR-W for LC incidence in Central Serbia (1999–2023) was 6.2 per 100,000, with a pronounced sex disparity (11.1 in men vs. 2.7 in women).

In comparison, GLOBOCAN data for 2022 revealed substantial regional and global variation in ASR-W for LC incidence. In Europe, the average ASR-W for LC incidence in 2022 was 2.7 per 100,000 population. The highest ASR-W for LC incidence (>3.6 per 100,000) was reported in Moldova (5.5), Romania (5.1), Hungary (4.5), Belarus (4.3), Poland (4.3), Bosnia and Herzegovina (4.2), Serbia (4.1), and Montenegro (4.0). Conversely, the lowest ASR-W for LC incidence in 2022 (<1.5 per 100,000) was observed in Switzerland (1.3) and in Nordic countries such as Iceland (1.1), Norway (1.0), Finland (0.9), and Sweden (0.7) [5,6,23,24].

Worldwide, the highest ASR-W for LC incidence (>2.5 per 100,000) was recorded in the Caribbean (4.3), Eastern Europe (3.4), Southern Europe (3.1), Western Asia (2.7), and South-Central Asia (2.6). On the other hand, the lowest ASR-W for LC incidence (<0.9 per 100,000) was reported in Micronesia (0.8), Middle Africa (0.8), and Western Africa (0.8) [5,6,23,24].

Globally, men (3.4 per 100,000) are diagnosed with LC 7 to 10 times more frequently than women (0.5 per 100,000) [6]. The most pronounced sex disparity was observed in Eastern Europe, where the ASR-W for LC incidence in men (7.2 per 100,000) was 14 times higher than in women (0.5 per 100,000), markedly exceeding the disparity recorded in Central Serbia (7.9 times). By comparison, in North America, the male ASR-W (3.2 per 100,000) is only four times that of females (0.8 per 100,000) [5,6,23,24].

According to our findings, the average ASR-W for LC mortality in Central Serbia (1999–2023) was 2.9 per 100,000, with a marked sex disparity (5.4 in men vs. 0.5 in women). In Europe, approximately 19,000 people died from LC in 2022, corresponding to an ASR-W for LC mortality of 1.2 per 100,000 population. The highest ASR-W for LC mortality in Europe (>2.3 per 100,000) was reported in Eastern Europe, particularly in Moldova (3.1), Romania (2.9), Montenegro (2.6), Bulgaria (2.5), and Belarus (2.3). Conversely, the lowest ASR-W for LC mortality (<0.5 per 100,000) was recorded in Luxembourg (0.5), Switzerland (0.3), and Nordic countries, including Finland (0.3), Norway (0.3), Sweden (0.3), and Iceland (0.1) [5,6,23,24].

Worldwide, in 2022, the highest ASR-W for LC mortality (>1.3 per 100,000) was recorded in the Caribbean (2.2), Eastern Europe (1.9), South-Central Asia (1.7), Northern Africa (1.5), Western Asia (1.4), and South America (1.4). In contrast, the lowest ASR-W for LC mortality (<0.4 per 100,000) was observed in Australia and New Zealand, specifically 0.4 per 100,000. An 8–10-fold higher mortality rate from LC in men (1.7 per 100,000) compared to women (0.2 per 100,000) has been reported, reflecting underlying incidence trends. This pattern is consistent with our findings, which showed a male-to-female mortality ratio of 10.8 [5,6,23,24].

### 4.2. Epidemiological Trends

The findings of this study provide a comprehensive analysis of long-term trends in LC incidence and mortality in Central Serbia. Over the past 25 years, LC has remained a significant public health concern among men, while its impact on women has been considerably lower.

In our analysis, LC mortality among men declined more rapidly (APC = −2.0%) than incidence (APC = −0.7%), suggesting that the observed mortality reduction is more likely attributable to advances in treatment and disease management, which typically yield more immediate and consistent effects than shifts in risk factor prevalence. This aligns with established epidemiological evidence showing that men are disproportionately affected due to greater exposure to key risk factors, such as tobacco use, alcohol consumption, and occupational carcinogen exposure [7,25,26,27]. Despite significant decreases in both incidence and mortality among men, trends among women warrant attention. While mortality remained relatively stable (APC = −0.7%, non-significant), a non-significant increase in incidence (APC = +0.9%) was observed, potentially reflecting emerging risk patterns. This upward trend in women may be associated with increased tobacco use among younger women, as reported in other European countries [7,25,26,27]. The highest incidence was observed in the 60–69 age group, consistent with findings from other countries where LC is more common in older populations [10]. Between 2016 and 2019, notable advancements were made in Serbia’s oncology infrastructure. These included the procurement of new radiotherapy equipment, the establishment of new centers and the renovation of existing ones, the acquisition of advanced radiological diagnostic tools, and the partial expansion of oncology clinic capacities.

According to the American Cancer Society, LC incidence in the United States has declined by approximately 2–3% annually, primarily due to reductions in smoking, with mortality trends following a similar pattern [28]. A study from Italy reported a significant decline in newly diagnosed head and neck cancer cases during the early phase of the COVID-19 pandemic, likely attributable to reduced access to healthcare services and patient-related delays [29]. These findings reflect potential diagnostic disruptions observed in Central Serbia.

In Europe, LC mortality has decreased across most countries, particularly during the past three decades [30]. After a steady increase from the 1950s to the 1970s, LC mortality among men stabilized in Western and Southern Europe in the early 1980s and in Central and Eastern Europe in the early 1990s. Among EU men, mortality remained relatively stable between 1980 and 1991 (APC = −0.5%), followed by a sharp decline of 3.3% annually from 1991 to 2012 [31]. During this period, ASR-W for mortality decreased from 4.7 per 100,000 in 1990–1991 to 2.5 per 100,000 in 2010–2011. The highest male mortality rates in 2010–2011 were observed in Hungary, Moldova, and Romania (over 6 per 100,000), whereas the lowest were in Finland, Norway, Sweden, and Switzerland (below 1 per 100,000) [31].

Among EU women, mortality remained stable at approximately 0.29 per 100,000 between 1980 and 1994, followed by a slight decline from 1994 to 2000–2001 (APC = −1.3%), reaching 0.23 per 100,000. Overall, LC mortality has followed a favorable downward trend across most of Europe, primarily attributed to reductions in tobacco use and, in Mediterranean countries, a decrease in alcohol consumption [31].

A retrospective analysis of real-world data from the US Centers for Disease Control and Prevention (CDC) suggests that LC mortality remained stable during the COVID-19 pandemic, with age-adjusted death rates ranging from 0.89 to 0.91 per 100,000 between 2018 and 2022. No major sex- or age-related differences were detected. Following a slight increase in 2020, mortality among individuals aged 85 and over rose significantly in 2021. By 2022, rates had returned to pre-pandemic levels (2018–2019), likely reflecting lower SARS-CoV-2 virulence and the relaxation of public health restrictions [32]. Following the World Health Organization’s declaration in May 2023 that COVID-19 no longer constituted a Public Health Emergency of International Concern, a decline in LC mortality rates was observed, likely due to earlier diagnosis and improved survival [33]. Diagnostic delays during the pandemic period adversely affected treatment outcomes.

### 4.3. Risk Factors

Multiple risk factors contribute to the development of LC, with tobacco smoking recognized as the most significant [34,35]. Substantial evidence dating back to the 1950s has established a strong association between smoking and head and neck cancers, with approximately 95% of LC patients in the United States identified as smokers [8,36]. Alcohol consumption also plays a critical role, and the synergistic effect of tobacco and alcohol may increase the risk of LC by up to 177-fold [37,38,39]. Other well-established or emerging risk factors include human papillomavirus (particularly HPV-16), Epstein–Barr virus (EBV), *Helicobacter pylori* infection, and gastroesophageal reflux disease (GERD) [34]. HPV is more frequently detected in younger, non-smoking patients and may influence prognosis [40,41,42]. Both EBV and HPV contribute to carcinogenesis through the expression of viral oncoproteins [43]. Recent studies have also highlighted a potential role of the upper aerodigestive tract microbiome in LC pathogenesis, with bacteria such as *Fusobacterium* implicated in mucosal transformation [44]. Additional risk factors include genetic predisposition, prior exposure to radiotherapy, immunosuppression, occupational exposure to carcinogens, air pollution, and unhealthy dietary habits, particularly low intake of fruits and vegetables [34].

The results of the 2019 national survey underscore the significant burden of tobacco and alcohol use in Serbia, with important implications for cancer prevention and broader non-communicable disease (NCD) control efforts [45]. Despite a gradual decline over the past two decades, tobacco use remains widespread. In 2019, 31.9% of individuals aged 15 and older reported either daily or occasional use of tobacco products, with higher prevalence observed among men (33.9%) compared to women (30.1%). The highest smoking rates were recorded in the 45–54 age group (41.3%), while concerning levels of tobacco use were also noted among adolescents aged 15–19 (14.4%) and individuals from low-income households (35.5%). Among smokers, those who smoked 20 or more cigarettes per day accounted for 15.8% [45].

A comparison with earlier data shows that the percentage of daily smokers decreased from 33.0% in 2000 to 27.1% in 2019. In men, the decrease was considerable, with the proportion dropping from 46.0% to 29.4%, whereas in women, the reduction was minimal, with a decline from 26.1% to 25.0%. Notably, the overall prevalence of tobacco smoking in Serbia (30.5%) remains significantly higher than the European Union average of 23%. These trends highlight persistent gender and socioeconomic disparities and reinforce the need for targeted tobacco control interventions [45].

Although the Law on the Protection of the Population from Exposure to Tobacco Smoke was adopted in Serbia in 2010, survey results indicate gaps in both enforcement and public compliance [46]. Only 30.1% of smokers reported receiving cessation advice from healthcare professionals, suggesting missed opportunities for brief interventions in clinical settings. Strengthening professional education and integrating smoking cessation counseling into routine primary care could improve individual outcomes and reduce population-level exposure to tobacco-related harm [45].

In parallel, alcohol consumption also represents a pressing public health concern. While 50.7% of the Serbian population reported abstaining from alcohol in 2019 (including 39.3% who had never consumed it), nearly half (49.3%) reported alcohol use in the preceding year. Daily alcohol consumption declined slightly from 3.3% in 2000 to 3.1% in 2019. This reduction was more pronounced among men (from 6.7% to 5.7%), while an increase was observed among women (from 0.3% to 0.7%) [45].

Importantly, daily alcohol use remains markedly more common among men, approximately eight times higher than in women, and is most prevalent among individuals with the lowest levels of education and those residing in suburban areas. In addition to chronic consumption, patterns of episodic heavy drinking were also documented. At least once a week, 1.7% of the population (3.2% of men and 0.3% of women) engaged in single-occasion risky drinking (≥60 g of pure ethanol), with a monthly prevalence of 10.9%. Although this rate is lower than the EU average (14.4%), it still represents a significant risk factor for acute and chronic health conditions, including cancers of the upper aerodigestive tract [45].

### 4.4. Study Strengths and Limitations

This study has several methodological limitations that should be considered when interpreting the findings. First, the quality and coverage of data from the Cancer Registry of Central Serbia may have varied over the study period. Incomplete case reporting, potential under registration, and regional disparities in data collection may have affected the accuracy of estimated incidence and mortality rates.

The Cancer Registry does not provide data on TNM staging (it includes only the clinical stage), treatment modalities, or patient survival; however, it does include data on the histopathological type and anatomical subsite of the tumor (glottis, supraglottis, subglottis). Additionally, there are no data on the average time from the onset of symptoms to the diagnosis, as the current health information system does not support the automated collection of such data, which limits the ability to assess the impact of early detection and therapeutic modalities on disease outcomes and survival. These limitations highlight significant gaps in the available clinical information and underscore the need for more comprehensive data collection. As a result, the ability to evaluate the impact of early detection and treatment strategies on disease outcomes and survival remains limited.

A major strength of our study lies in the relative completeness and consistency of mortality data for LC, which are officially recorded through death certificates and systematically coded by cause of death. This makes mortality statistics more standardized and less prone to underreporting, as death is a distinct and final event. Although challenges such as ill-defined or unspecified causes of death still exist, it is less likely that a death will be entirely omitted from official records.

In contrast, incidence data are subject to greater limitations. The completeness of Cancer registry data may be affected by disparities in access to healthcare, leading to undiagnosed or unreported cases. The use of multiple data sources (e.g., hospitals, pathology reports, outpatient facilities) increases the risk of data entry errors, case duplication, or omissions. Diagnostic uncertainty, especially in early-stage disease, and variability in reporting practices and staff training can further reduce data quality. Additionally, coding inconsistencies and difficulties in accurately defining the population at risk—particularly in regions with high migration—may compromise the precision of incidence rates. Despite these limitations, incidence data are indispensable for assessing the true burden of cancer, monitoring long-term trends, and guiding public health planning and resource allocation.

Another notable limitation is the absence of individual-level data on key risk factors such as tobacco use, alcohol consumption, and HPV status. Without such data, causal inferences regarding observed trends must be made with caution, as potential shifts in population-level exposure cannot be directly assessed.

Advancements in diagnostic and therapeutic practices over the past two decades—including improved access to endoscopic evaluation, advanced imaging techniques, and updated treatment protocols—may have contributed to earlier detection and reduced mortality rates. While these developments likely reflect genuine improvements in healthcare delivery, they may also confound interpretation of trends by influencing disease classification and outcomes independently of changes in actual incidence or mortality.

During the reporting of malignant tumors in the analyzed period, only the ICD-10 classification was used for coding the anatomical site and tumor morphology, as implemented through the CanReg cancer registry software [47].

Finally, the geographic scope of the analysis was limited to Central Serbia. Data from other administrative regions, including Vojvodina and Kosovo and Metohija, were not included. Consequently, the generalizability of these findings to the entire Serbian population is restricted, and national-level conclusions should be drawn with caution.

Despite these limitations, the study provides important insights into the long-term epidemiological dynamics of LC in Central Serbia. It underscores the need for future research that integrates broader geographic coverage, clinical and pathological tumor features, and individual-level risk factor data.

### 4.5. Public Health Relevance and Prevention Opportunities

Organized early detection for LC has not been implemented in the Republic of Serbia. Consequently, prevention efforts are limited to controlling modifiable risk factors, addressed by broader public health strategies. The following oncology-related prevention programs have been introduced in chronological order: (1) Tobacco Control Strategy [48]; (2) The National Program for the Prevention of Cervical Cancer [49]; (3) Strategy for the Prevention and Control of Non-Communicable Diseases in Serbia [50]; (4) National Program for the Prevention of Harmful Use of Alcohol and Alcohol-Related Disorders [51]; (5) Strategy of Public Health in the Republic of Serbia, 2018–2026 [52]; (6) Program for the Improvement of Cancer Control in the Republic of Serbia for the Period 2020–2022 [53].

Since 2022, HPV vaccination has been included in the recommended national immunization program. However, its potential effect on the incidence of LC remains unclear, as the causal role of HPV in the pathogenesis of LC has not yet been definitively established [40,41,42].

Future research, with an emphasis on prospective study designs, is essential to elucidate the risk factors associated with the occurrence of this malignancy. Particular attention should be given to sex- and age-specific analyses, which would support the further refinement of prevention measures. Serbian rulebook on healthcare quality indicators, which was adopted in 2021, will enable the collection of two new indicators: (1) the average waiting time for the initiation of chemotherapy (including biologic systemic therapy), and (2) the proportion of patients who start radiotherapy within 28 days after indication [54]. Moreover, reinforcing cancer registries and implementing early detection programs may improve disease surveillance, facilitate timely diagnosis, and support more effective allocation of healthcare resources. Strengthening the cancer information system, investing in diagnostic infrastructure, and updating clinical protocols are public health priorities essential for reducing the burden of LC in Serbia.

## 5. Conclusions

Laryngeal cancer remains a significant public health concern in Central Serbia, particularly among men, where it ranks among the leading cancer types by incidence and mortality. Between 1999 and 2023, the incidence declined among men, but slightly increased among women, while mortality decreased in the male population and remained stable among women.

Efforts to reduce the disease burden should focus on early diagnosis, timely treatment, and health education that promotes risk reduction and symptom awareness, particularly among older adults. Strengthening the enforcement of tobacco control legislation, expanding access to cessation services, and improving health literacy—especially among vulnerable groups—should be prioritized.

Although HPV vaccination is a key tool in the prevention of several anogenital and oropharyngeal cancers, its potential role in LC prevention remains to be further elucidated through future anatomical-site-specific research.

To ensure timely response and intervention, continuous epidemiological surveillance and improved harmonization of national cancer data systems are essential for monitoring trends, identifying high-risk populations, and evaluating the effectiveness of public health strategies.

## Figures and Tables

**Table 1:** Crude and standardized * incidence rates (per 100,000) for laryngeal cancer by sex, Central Serbia, 1999–2023.

Year	Crude Incidence Rate			Standardized Incidence Rate		
	Men	Women	Total	Men	Women	Total
1999	17.3	1.4	9.4	10.9	0.9	5.9
2000	19.8	1.9	10.8	12.3	1.1	6.7
2001	21.1	2.2	11.6	13.1	1.3	7.2
2002	21.0	2.1	11.6	12.9	1.2	7.0
2003	19.8	2.6	11.2	12.1	1.6	6.8
2004	18.8	2.2	10.5	11.4	1.3	6.4
2005	18.5	2.1	10.3	11.4	1.3	6.4
2006	22.0	2.4	12.2	13.5	1.4	7.4
2007	19.5	2.8	11.2	11.6	1.5	6.6
2008	18.8	2.6	10.7	11.2	1.6	6.4
2009	19.4	2.0	10.7	11.6	1.2	6.4
2010	19.5	3.2	11.1	11.6	1.8	6.7
2011	18.9	3.1	10.8	10.9	1.8	6.4
2012	17.8	2.0	9.7	10.1	1.0	5.6
2013	18.3	3.0	10.5	10.0	1.5	5.8
2014	18.1	2.3	10.2	9.7	1.1	5.4
2015	20.3	2.5	11.4	10.9	1.3	6.1
2016	18.0	2.1	9.9	9.3	1.1	4.9
2017	17.3	3.0	10.0	9.0	1.4	5.0
2018	19.9	3.4	11.4	10.2	1.7	5.6
2019	21.6	3.6	12.4	10.9	1.8	6.0
2020	18.6	3.2	10.7	8.9	1.3	4.8
2021	22.8	3.6	12.9	11.2	1.6	6.0
2022	20.9	3.7	12.1	10.1	1.5	5.5
2023	24.8	3.6	13.9	11.9	1.6	6.4
Average **	19.7	2.7	11.1	11.1	1.4	6.2

* Standardized rates according to the world population, ** Average incidence rate (1999–2023).

**Table 2:** Average age-specific and standardized incidence rates (per 100,000) ** for laryngeal cancer by sex: Joinpoint Trend Analysis, Central Serbia, 1999–2023.

Men						Women			
**Age Group** **(Years)**	Incidence Rate	Period	APC (95% CI)	AAPC (95% CI)	Age Group (Years)	Incidence Rate	Period	APC (95% CI)	AAPC (95% CI)
0–39	0.5	1999–2023	−5.6 * (−8.7, −2.5)	−5.6 * (−8.7, −2.5)	0–49	0.7	1999–2023 1999–2002 2002–2023	62.9 * (6.4, 277.3) −7.7 * (−11.4, −5.4)	−0.9 (−4.8, 4.2)
40–49	10.4	1999–2023 1999–2010 2010–2019 2019–2023	0.1 (−3.1, 5.4) −14.7 * (−29.5, −10.5) 19.4 * (2.5, 63.4)	−2.8 * (−4.4, −1.2)					
50–59	39.4	1999–2023	−2.6 * (−3.5, −1.9)	−2.6 * (−3.5, −1.9)	50–59	4.6	1999–2023	0.5 (−1.4, 2.6)	0.5 (−1.4, 2.6)
60–69	61.1	1999–2023	0.9 * (0.1, 1.8)	0.9 * (0.1, 1.8)	60–69	7.4	1999–2023	2.7 * (1.6, 4.5)	2.7 * (1.6, 4.5)
70+	47.9	1999–2023 1999–2017 2017–2023	−0.4 (−5.0, 0.8) 7.8 * (1.6, 25.4)	1.6 * (0.2, 2.6)	70+	5.1	1999–2023	2.8 * (1.5, 5.0)	2.8 * (1.5, 5.0)
Average Standardized Rate	11.1	1999–2023 1999–2017 2017–2023	−1.6 * (−3.9, −1.1) 2.0 (−0.7, 10.5)	−0.7 * (−1.4, −0.2)	Average Standardized Rate	1.4	1999–2023	0.9 (−0.2, 2.0)	0.9 (−0.2, 2.0)

APC—Annual Percent Change, AAPC—Average Annual Percent Change, 95% CI—95% Confidence Interval, * APC/AAPC is significantly different from 0 at the α = 0.05 significance level, ** Standardized rates according to the world population.

**Table 3:** Age-specific incidence rates (per 100,000) for laryngeal cancer by sex, Central Serbia, 1999–2023.

Age (Years)	20–29		30–39		40–49		50–59		60–69		70+	
Sex	M	W	M	W	M	W	M	W	M	W	M	W
Years												
1999	0.0	0.0	1.1	0.0	13.0	0.9	42.9	4.5	53.4	5.4	40.8	1.5
2000	0.0	0.6	1.2	0.0	12.6	0.8	47.2	3.6	64.8	4.3	47.5	5.9
2001	0.0	0.0	1.4	0.3	13.8	3.5	58.2	3.4	63.6	6.2	44.9	3.3
2002	0.6	0.0	1.4	0.3	15.4	2.2	51.1	3.4	63.6	4.8	46.0	5.7
2003	0.0	0.0	2.0	0.6	13.4	4.0	52.1	3.4	49.3	7.6	52.0	4.2
2004	0.5	0.2	0.9	0.3	15.1	3.0	43.9	4.6	54.2	5.6	51.8	3.4
2005	0.2	0.0	0.3	0.0	17.6	3.1	42.2	5.6	52.8	4.5	42.0	2.7
2006	0.6	0.2	1.2	0.0	18.4	2.4	47.2	5.4	68.8	7.4	51.2	3.0
2007	0.8	0.0	1.7	0.8	13.8	2.2	39.6	5.8	57.3	5.6	52.2	5.9
2008	0.0	0.0	1.4	0.6	12.0	1.7	48.1	5.2	52.4	9.2	40.3	3.7
2009	0.0	0.6	1.1	0.0	14.0	2.5	44.0	3.5	60.1	4.9	43.2	3.4
2010	0.0	0.0	0.6	0.6	12.8	2.0	49.2	7.1	60.6	10.0	34.6	4.7
2011	0.0	0.6	0.8	0.2	10.0	2.0	39.6	6.0	63.0	8.4	42.5	7.0
2012	0.0	0.0	0.2	0.0	14.4	1.7	34.2	3.3	53.6	5.6	41.0	3.4
2013	0.3	0.0	0.6	0.0	7.9	1.4	35.8	6.0	57.2	7.0	42.0	7.2
2014	0.3	0.0	1.1	0.2	6.2	0.8	33.6	4.8	58.6	6.3	45.4	3.2
2015	0.3	0.3	0.6	0.0	8.2	2.2	35.1	2.8	67.2	7.8	47.6	4.5
2016	0.0	0.0	0.3	0.3	6.6	0.8	28.5	4.9	57.2	5.2	47.1	3.1
2017	0.3	0.0	0.3	0.3	5.7	1.1	30.2	4.2	57.4	8.3	38.7	6.7
2018	0.7	0.3	0.8	0.3	2.8	2.0	31.5	5.1	70.8	10.1	45.6	5.6
2019	0.0	0.0	0.9	0.0	3.4	2.0	35.1	6.3	72.5	10.1	52.7	6.3
2020	0.0	0.0	0.0	0.0	5.3	0.3	23.0	2.9	51.0	11.0	64.4	7.1
2021	0.0	0.0	0.3	0.0	4.7	0.6	33.3	5.9	70.2	9.4	67.0	8.2
2022	0.0	0.0	0.3	0.3	5.4	0.6	30.3	3.5	64.1	10.5	54.6	9.3
2023	0.0	0.0	0.3	0.3	7.7	0.6	28.5	4.7	83.8	10.7	62.8	7.5
Average *	0.2	0.1	0.8	0.2	10.4	1.8	39.4	4.6	61.1	7.4	47.9	5.1

M—Men, W—Women, * Average incidence rate (1999–2023).

**Table 4:** Crude and standardized * mortality rates (per 100,000) for laryngeal cancer by sex, Central Serbia, 1999–2023.

Year	Crude Mortality Rate			Standardized Mortality Rate		
	Men	Women	Total	Men	Women	Total
1999	11.7	1.0	6.4	7.0	0.5	3.8
2000	11.8	1.0	6.4	7.1	0.5	3.8
2001	10.5	1.0	5.8	6.2	0.5	3.4
2002	10.1	1.1	5.6	5.9	0.6	3.2
2003	11.7	0.9	6.3	6.7	0.5	3.6
2004	10.5	1.1	5.8	6.0	0.6	3.3
2005	10.3	1.2	5.8	5.6	0.6	3.1
2006	10.4	1.1	5.8	5.9	0.5	3.2
2007	10.5	1.1	5.8	5.9	0.5	3.2
2008	10.2	1.4	5.8	5.5	0.7	3.1
2009	11.2	0.8	6.0	6.1	0.3	3.2
2010	9.8	1.3	5.6	5.0	0.6	2.8
2011	10.8	1.2	6.0	5.9	0.5	3.2
2012	10.7	1.4	6.0	5.5	0.6	3.0
2013	9.6	1.2	5.4	4.9	0.5	2.7
2014	9.6	0.9	5.2	4.9	0.3	2.6
2015	9.6	1.2	5.4	4.7	0.5	2.6
2016	9.6	1.3	5.3	4.7	0.5	2.4
2017	10.6	0.9	5.6	5.2	0.4	2.6
2018	9.9	1.3	5.5	4.8	0.6	2.5
2019	10.6	0.8	5.6	4.9	0.3	2.4
2020	9.5	1.3	5.3	4.3	0.4	2.2
2021	9.3	1.1	5.1	4.2	0.4	2.1
2022	8.8	1.6	5.1	4.1	0.6	2.2
2023	9.1	1.1	5.0	4.0	0.4	2.1
Average **	10.3	1.1	5.7	5.4	0.5	2.9

* Standardized rates according to the world population, ** Average mortality rate (1999–2023).

**Table 5:** Average age-specific and standardized mortality rates (per 100,000) ** for laryngeal cancer by sex: Joinpoint Trend Analysis, Central Serbia, 1999–2023.

Men						Women			
Age Group (Years)	Incidence Rate	Period	APC (95% CI)	AAPC (95% CI)	Age Group (Years)	Incidence Rate	Period	APC (95% CI)	AAPC (95% CI)
0–49	1.3	1999–2023	−4.9 * (−7.2, −3.8)	−4.9 * (−7.2, −3.8)	0–59	0.7	1999–2023	−1.2 (−3.5, 0.5)	−1.2 (−3.5, 0.5)
50–59	17.1	1999–2023	−2.5 * (−4.5, −1.1)	−2.5 * (−4.5, −1.1)					
60–69	29.9	1999–2023	−1.3 * (−1.9, −0.6)	−1.3 * (−1.9, −0.6)	60–69	4.6	1999–2023	−0.4 (−2.3, 1.3)	−0.4 (−2.3, 1.3)
70+	35.7	1999–2023 1999–2010 2010–2015 2015–2020 2020–2023		−0.8 * (−1.5, −0.3)	70+	7.4	1999–2023	1.5 * (0.3, 3.1)	1.5 * (0.3, 3.1)
			1.0 * (0.1, 2.7)						
			−4.6 * (−10.0, −1.8)						
			4.8 * (1.9, 11.8)						
			−9.4 * (−19.8, −4.2)						
Average Standardized Rate	5.4	1999–2023	−2.0 * (−2.5, −1.6)	−2.0 * (−2.5, −1.6)	Average Standardized Rate	1.4	1999–2023	−0.7 (−1.9, 0.4)	−0.7 (−1.9, 0.4)

APC—Annual Percent Change, AAPC—Average Annual Percent Change, 95% CI—95% Confidence Interval, * APC/AAPC is significantly different from 0 at the α = 0.05 significance level, ** Standardized rates according to the world population.

**Table 6:** Age-specific mortality rates (per 100,000) for laryngeal cancer by sex, Central Serbia, 1999–2023.

Age (Years)	20–29		30–39		40–49		50–59		60–69		70+	
Sex	M	W	M	W	M	W	M	W	M	W	M	W
Years												
1999	0.0	0.0	0.2	0.0	7.8	0.9	20.6	1.2	40.9	2.7	36.2	3.7
2000	0.0	0.0	0.3	0.2	7.1	0.7	23.4	1.6	37.4	1.6	38.4	2.9
2001	0.0	0.0	0.6	0.0	4.6	0.4	20.8	1.8	32.8	2.4	35.2	3.3
2002	0.0	0.0	0.0	0.0	3.7	0.4	24.2	1.8	31.3	3.4	29.5	3.4
2003	0.0	0.0	0.0	0.6	4.1	0.7	25.2	1.0	34.4	2.0	40.3	2.2
2004	0.0	0.0	0.3	0.0	5.3	0.0	18.2	2.2	31.8	2.2	38.0	3.2
2005	0.0	0.0	0.3	0.0	3.2	1.0	20.1	1.7	28.0	2.2	39.6	3.8
2006	0.0	0.3	0.0	0.0	5.5	0.5	19.4	1.2	31.2	2.4	34.4	4.0
2007	0.0	0.0	0.3	0.0	5.5	0.6	20.1	1.8	28.4	3.0	36.6	2.7
2008	0.0	0.0	0.3	0.0	3.6	0.3	15.6	2.4	30.2	4.6	39.0	3.4
2009	0.0	0.0	0.0	0.0	5.2	0.0	19.4	1.2	29.4	1.6	40.9	2.8
2010	0.0	0.0	0.0	0.3	1.8	0.0	16.4	1.6	26.8	3.7	39.8	4.0
2011	0.0	0.0	0.0	0.0	3.8	0.3	18.4	1.2	34.0	2.4	36.2	4.8
2012	0.0	0.0	0.2	0.0	4.4	0.8	31.9	2.4	32.0	1.2	35.2	4.6
2013	0.0	0.0	0.2	0.0	3.6	0.6	15.0	1.0	25.0	2.0	33.8	4.2
2014	0.0	0.0	0.6	0.0	2.6	0.0	15.2	0.6	26.1	2.4	32.8	3.2
2015	0.0	0.0	0.0	0.0	1.2	0.3	15.2	1.0	29.2	3.0	30.2	4.1
2016	0.0	0.0	0.3	0.0	2.9	0.3	11.6	0.8	28.3	2.7	32.9	4.9
2017	0.0	0.0	0.0	0.3	2.6	0.0	14.5	0.3	30.0	2.4	36.1	3.4
2018	0.0	0.0	0.6	0.0	2.0	0.8	12.9	1.7	29.7	2.5	32.2	3.6
2019	0.0	0.0	0.3	0.0	0.6	0.0	13.4	0.0	27.3	2.0	41.4	3.2
2020	0.0	0.0	0.3	0.0	2.0	0.3	6.8	0.6	28.3	1.8	36.1	5.6
2021	0.0	0.0	0.0	0.0	2.0	0.3	9.3	1.2	23.3	1.8	37.7	4.1
2022	0.0	0.0	0.0	0.0	2.6	0.3	10.8	2.1	22.5	3.4	30.3	4.8
2023	0.0	0.0	0.0	0.0	0.9	0.0	7.8	0.6	29.2	2.4	30.1	4.0
Average *	0.0	0.0	0.2	0.1	3.5	0.4	17.1	1.3	29.9	2.5	35.7	3.8

M—men, W—women, * Average age-specific mortality rate (1999–2023).

## Data Availability

Relevant data are available from the corresponding author upon reasonable request.
